# Chocolate Eaters Do Not Necessarily Win a Nobel Prize—Authors in Special Issues Do Not Necessarily Publish Lower Quality Papers

**DOI:** 10.3389/ijph.2023.1606967

**Published:** 2023-12-22

**Authors:** Nino Künzli, Christopher Woodrow, Anke Berger, Katarzyna Czabanowska, Licia Iacoviello, Raquel Lucas, Andrea Madarasova Geckova, Sonja Merten, Olaf von dem Knesebeck, Sarah Mantwill, Salvatore Panico, Lyda Osorio, Ana Isabel Ribeiro, Paolo Chiodini, L. Suzanne Suggs, Jean Coulibaly

**Affiliations:** ^1^ Swiss Tropical and Public Health Institute, Allschwil, Switzerland; ^2^ University of Basel, Basel, Switzerland; ^3^ Swiss School of Public Health, Zürich, Switzerland; ^4^ Department of International Health, Care and Public Health Research Institute (CAPHRI), FHML, Maastricht University, Maastricht, Netherlands; ^5^ Department of Epidemiology and Prevention, IRCCS Neuromed, Pozzilli, Italy; ^6^ Department of Medicine and Surgery, LUM University, Casamassima, Italy; ^7^ Epidemiology Research Unit, Institute of Public Health, University of Porto, Porto, Portugal; ^8^ Department of Health Psychology and Research Methodology, Pavol Jozef Šafárik University in Košice, Košice, Slovakia; ^9^ Institute of Medical Sociology, University of Hamburg, Hamburg, Germany; ^10^ Faculty of Health Sciences and Medicine, University of Lucerne, Lucerne, Switzerland; ^11^ School of Medicine, Federico II University, Naples, Italy; ^12^ Centro Internacional de Entrenamiento e Investigaciones Médicas Universidad del Valle, Cali, Colombia; ^13^ Medical Statistics Unit—University of Campania “Luigi Vanvitelli”, Naples, Italy; ^14^ Institute of Communication and Public Policy, Faculty of Communication, Culture, and Society, Università della Svizzera Italiana, Lugano, Switzerland; ^15^ Unité de Formation et de Recherche Biosciences, Université Félix Houphouët-Boigny, Abidjan, Côte d’Ivoire

**Keywords:** editorial policies, science funding policies, peer review, special issues, regular issues

We, the editors of the independent, non-profit journals of the Swiss School of Public Health (SSPH+), meet every Fall for strategic planning. At Public Health Reviews (PHR) and International Journal of Public Health (IJPH), all editorial strategies and peer-review decisions have remained under the sole control of academically rooted scientists since the journals were founded, 50 and 102 years ago respectively. With the move to Gold Open Access (OA), SSPH+ continued to contract with a professional publisher to ensure up to date technical, legal, and ethical publishing standards.

One of our responsibilities is the strategic shaping of the content of the journals. In line with public research funding agencies that support both investigator-initiated research and research based on special calls for proposals on specific topics, our journals feature both investigator-driven regular publications and publications related to calls for papers on relevant topics that are published as Special Issues (SI). For example, early on in the COVID-19 pandemic, we identified the need for OA-published research on the associated mental health effects. Our call provided a platform that was highly appreciated by the science community with 60 articles successfully having passed independent peer-review, including well-cited papers [1, 2]. Special Issues are a formidable strategic tool for editors and authors to foster relevant science that is timely and deserving of being consolidated in focused issues of journals.

However, SI currently face a challenge from unjustified criticism of the SI-based growth of genuine OA journals. A non-peer reviewed paper provides a sobering example of this criticism [3]. It appropriately addresses the enormous strain on scientists related to the growth of publications. The two SSPH+ journals also experience the consequences of this strain, including difficulties in finding reviewers and long peer review processes [4]. However, we disagree with the implicit messages of the article as it is based on inadequate methodology. In particular, the authors simply aggregate all publications of 7 years at the publisher level and compare purely quantitative indicators across publishers. Though the authors acknowledge that the chosen indicators provide no insights into quality, narratives presented in the paper clearly promote the incorrect conclusion that articles in SI are both the cause of the strain and of inferior quality compared to regular articles. With the sole focus on publishers, Hanson et al. [3] do not open Pandora’s box of quality. We are ready to do so.

Apart from authors’ responsibilities, the quality of published articles is determined by the quality of the peer-review, which depends on the editor governance model of a journal. Editors-in-Chief and Handling Editors decide what to promote for peer-review and what to accept or reject for publication, based on the reviewer’s feedback and their own expertise. Insufficient qualification for this quality assurance might be the biggest threat to peer review quality. In the traditional model—like in the SSPH+ journals—independent science-based editors are the prime constituencies that guarantee quality as they decide what gets published. Instead, in the non-academic editor model, hired publisher staff not active in science have taken up this role. This latter model exists both for SI and regular publications, and for journals owned by traditional and new OA publishers. Given that all kinds of conflicts of interest may jeopardize quality (and influence quantity), a key question is how independent the decision-making editors are from the publishing business and other non-scientific interests or possible conflicts. In fact, many of the ten publishers considered in Hanson et al. [3] publish both independent “society journals,” and publisher-owned journals. The former might assign all editorial decisions to independent scientists whereas in the latter, both editorial governance models exist.

And to open the box wider: some journals, both independent and publisher-owned ones, may adopt different editorial decision models for SI versus regular submissions. Many journals—like ours—apply the same rigorous peer review to regular and SI submissions. We also support guest editors joining our experienced journal editors (Editor-in-Chief or Senior Editor) who prescreen submissions and check final peer review decisions for quality assurance. Conversely, some journals may weaken the review process of SI e.g., with Guest Editors asking “friends and family” for submissions and/or to review. Moreover, some publishers—with or without rigorous peer review—may focus on growing the revenues of their own journals through the acquisition of as many papers as possible, using non-academic staff, supported by AI, to identify “hot topics,” leaders, and authors for SI. We all—and our spam filters—regularly receive such invitations from non-academic staff.

The multifaceted Pandora’s box confirms two points. First,—as in the case of regular submissions—scientists should only submit to SI that use rigorous peer review, led by scientific editors, who are experts in the field. Second, quantitative indicators aggregated at the level of ten publishers cannot capture quality nor distinguish between articles published regularly versus in SI. Thus, the naïve comparison of Hanson et al. [3] cannot support the claim that articles in SI are of lower quality than regular publications. This is as odd as using the correlation between the national chocolate consumption and the *per capita* Nobel Prize Laureates to promote that eating chocolate boosts peoples’ scientific skills [5].

Hanson et al. [3] provide one quantitative indicator that is used as a proxy for quality, namely, the observation of shorter and more homogenous average peer review times for SI compared to regular publications. The narrative is that quicker peer review is an indicator of lower SI rigor and quality. The figure provided (Fig3supp2 in [3]), a graph that shows average SI and regular publication review times for eight publishers across 7 years, leads us to entirely different conclusions. In the vast majority of all data points (87%), “turnaround time” is clearly longer than 2 months. The far more important question is why almost 80% of all the average “turnaround times”, both for SI and regular publications, lie between a lengthy 100 and 180 days. The observed 1–2 weeks shorter duration of the still long SI peer reviews matches our experience with editing SI. Peer review management tends to be more structured and targeted in SI (e.g., with well prepared calls, submission deadlines, and identification of relevant, topic-based scientists for potential peer review). To define reduced turnaround times as “suspicious” would require a quality-oriented analysis, rather than an over-interpretation of aggregated mean values of quantitative indicators. Instead of spreading collective suspicion against “faster” turnarounds in SI, scientists engaged as editors or reviewers welcome solutions to shorten average peer review cycles, for both SI and regular submissions. Failure on this front will further increase the flood—a strain in itself—of pre-print publications where authors opt for speed instead of quality peer-review.

Why do we care about this not reviewed paper? Surprisingly, in sharing the anti-SI narrative of Hanson et al. [3], a prime public funder of Swiss research will stop funding APCs for articles published in SI [6]. Although usually dedicated to evidence and quality, the foundation is adopting a policy that lacks meaningful evaluation of quality. Whilst the rationale provided for the policy collectively discredits science published in SI, the issue of whether grantees’ articles published in SI are of lower quality than their regular articles has not been addressed.

It sets a dangerous and unscientific precedent for PHR and IJPH that an external constituency and a public agency attempts to influence the editorial strategy of independent science-driven non-profit journals. We will defend the principle of editorial freedom and continue to strategically shape the content of our journals. As we object to the discrediting of honest scientists who publish good papers in SI, we continue to welcome the submission of quality papers to all of our calls. Moreover, we continue to hope that public funding agencies promote solutions for independent, “diamond” OA publishing, guaranteeing that articles accepted via rigorous peer review are published free of charge for authors and readers, either as SI or as regular articles.

Meanwhile, we will enjoy consuming Swiss chocolate during our annual strategic discussions on public health science topics to be featured in high quality Special Issues of both PHR and IJPH.
